# Physical activity and exercise in primary open-angle glaucoma: a scoping review

**DOI:** 10.3389/fphys.2026.1834649

**Published:** 2026-07-15

**Authors:** Valentin Schuhmann, Janine Küttel, Jan Van Eijgen, Konstantin Gugleta, Henner Hanssen

**Affiliations:** 1 Department of Sports, Exercise and Health, Division of Sports and Exercise Medicine, Medical Faculty, University of Basel, Basel, Switzerland; 2 Department of Ophthalmology, University Hospitals Leuven, Leuven, Belgium; 3 Research Group of Ophthalmology, Department of Neurosciences, Katholieke Universiteit Leuven, Leuven, Belgium; 4 Department of Ophthalmology, University of Basel, Basel, Switzerland

**Keywords:** disease progression, intraocular pressure, neurodegeneration, ocular perfusion, systemic health, vascular dysregulation

## Abstract

**Introduction:**

Primary open-angle glaucoma (POAG) is a leading cause of irreversible blindness worldwide. Although intraocular pressure (IOP) reduction remains the cornerstone of management, increasing attention has focused on physical activity (PA) and structured exercise as potential adjunct strategies. Exercise induces systemic physiological adaptations, including changes in systemic vascular health, autonomic regulation, and perfusion dynamics, which may influence glaucoma pathophysiology and ocular health beyond IOP-dependent pathways.

**Methods:**

This scoping review was conducted in accordance with PRISMA-ScR guidelines. MEDLINE, Embase, and SPORTDiscus were systematically searched from database inception to 13 June 2024. Eligible studies investigated associations or effects of PA or exercise on POAG-related outcomes, including ophthalmological parameters, systemic vascular markers, and patient-reported outcomes. All study designs were considered. Data were extracted and synthesized narratively across predefined clinical domains: incidence, progression, disease severity, and quality of life.

**Results:**

Eighteen studies met the inclusion criteria (14 observational, 4 randomized controlled trials). Aerobic exercise interventions were consistently associated with short-term reductions in IOP and increases in ocular perfusion pressure. Observational evidence suggested that higher habitual PA was associated with lower glaucoma prevalence, reduced incidence, and slower disease progression. However, findings across studies were heterogeneous, and no study directly evaluated disease severity or vision-related quality of life. Methodological limitations included inconsistent outcome definitions, inadequate patient characterization, reliance on self-reported PA, and short follow-up durations.

**Conclusion:**

Current evidence suggests that higher levels of PA are associated with more favorable POAG-related outcomes, potentially mediated through both IOP-dependent and systemic vascular mechanisms affecting ocular perfusion. However, causal inference remains limited. Future research should prioritize adequately powered, long-term randomized trials incorporating objective PA assessment and comprehensive ophthalmological and patient-reported outcomes to clarify the role of exercise in glaucoma prevention and management.

## Graphical Abstract

**Figure:**
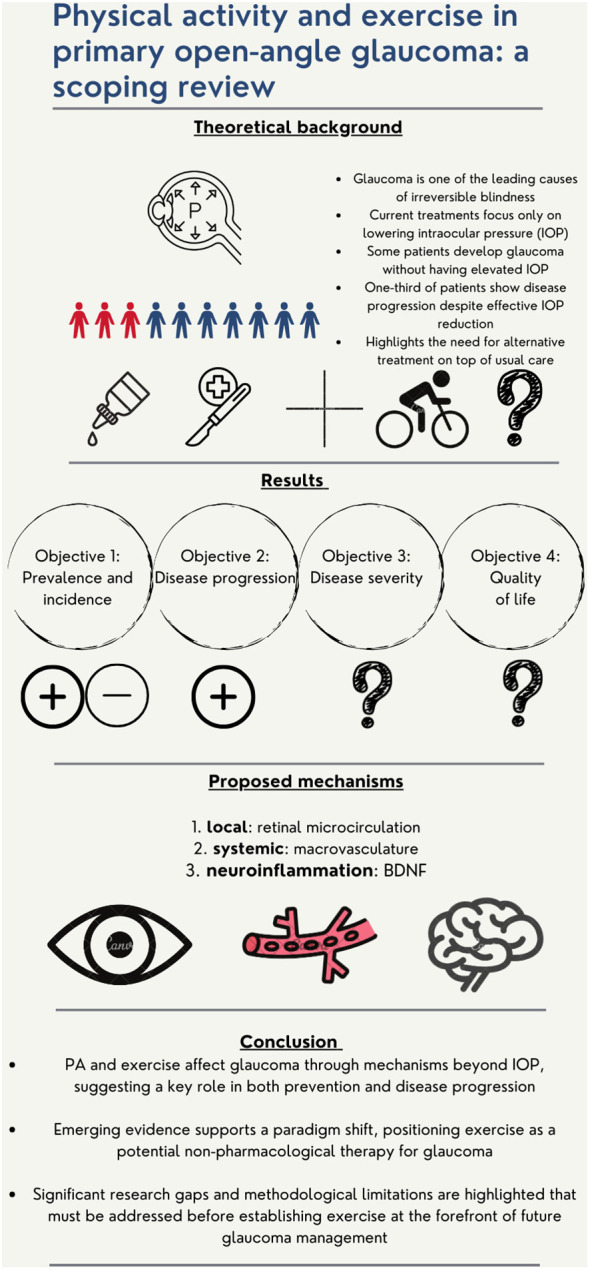

## Introduction

Glaucoma is a chronic, multifactorial optic neuropathy characterized by progressive loss of retinal ganglion cells (RGCs) and their axons, ultimately leading to irreversible visual field loss (Allison et al., 2020). It represents a major public health concern, being the second leading cause of blindness worldwide (Allison et al., 2020). Currently, more than 80 million people are affected, with projections estimating an increase to over 110 million by 2040, largely driven by population ageing (Tham et al., 2014). The gradual decline in visual function substantially impairs independence and quality of life (Thomas et al., 2015).

Primary open-angle glaucoma (POAG) is the most prevalent subtype, accounting for approximately three-quarters of all glaucoma cases. It is commonly classified as high-tension glaucoma (HTG) and normal-tension glaucoma (NTG) based on untreated IOP (Harasymowycz et al., 2016; Wagner et al., 2022). POAG, namely HTG is defined by elevated IOP with progressive optic nerve damage, whereas NTG involves statistically normal IOP with progressive optic neuropathy (Harasymowycz et al., 2016). The primary goal of glaucoma management is to slow structural and functional deterioration while preserving vision-related quality of life (Weinreb and Khaw, 2004; Harasymowycz et al., 2016; Garg and Gazzard, 2020).

While elevated IOP is the most important and only modifiable therapeutic target to date, disease progression may occur even when IOP is adequately controlled (Leske et al., 2003; Susanna et al., 2015; van Eijgen et al., 2024). Therefore, relying solely on IOP for risk stratification and diagnostic purposes to monitor treatment efficacy does not appear to be sufficient. In addition to elevated IOP, several systemic and vascular factors are implicated in glaucoma pathophysiology. These include age, myopia, genetic predisposition, smoking, and abnormalities in blood pressure regulation (Kumar and Ou, 2023; Zhang et al., 2024). Cardiometabolic risk factors such as obesity, hypertension, dyslipidaemia, and hypertriglyceridaemia have also been associated with glaucoma (Lin et al., 2010; Kreft et al., 2019; Talaat et al., 2021; Wändell et al., 2022). Large population-based data indicate a higher prevalence of cardiovascular comorbidities in glaucoma patients, and glaucoma itself may represent an independent risk factor for incident cardiovascular disease (Choi et al., 2023). These observations support the concept that vascular dysregulation, endothelial dysfunction, oxidative stress, and atherosclerotic processes may contribute to retinal nerve fibre layer damage and disease progression (Benoist d’Azy et al., 2016; Dascalu et al., 2021; Melgarejo et al., 2023). Moreover, disturbances in 24-hour blood pressure profiles, including nocturnal hypotension, hypertension, and increased variability, have been linked to POAG prevalence and progression (Van Eijgen et al., 2024).

Given that a considerable proportion of patients experience progression despite adequate IOP control, there is growing interest in complementary strategies that target IOP-independent mechanisms. Lifestyle-based and neuroprotective approaches are increasingly being explored as adjunctive interventions. Among these, physical activity (PA) has received particular attention. Regular PA is known to improve cardiometabolic and vascular health, reduce systemic inflammation, and exert neuroprotective effects across several neurodegenerative conditions (Zhu et al., 2018; Tribble et al., 2021; Chu-Tan et al., 2022; Kumar and Ou, 2023; Zhang et al., 2024). Conversely, physical inactivity has been associated with increased oxidative stress, systemic inflammation, and accelerated neurodegeneration (Chu-Tan et al., 2022; Kumar and Ou, 2023; Zhang et al., 2024). These mechanisms may also be relevant in the context of glaucoma.

An emerging body of literature suggests that PA and structured exercise may influence glaucoma-related outcomes, including IOP, ocular perfusion, disease incidence, and progression. However, the available evidence is heterogeneous, encompassing different glaucoma patient labeling, intervention protocols and outcome measures. Furthermore, the number of randomized controlled trials is limited, and most data is derived from observational studies. Previous reviews have typically focused on single aspects of the relationship between exercise and glaucoma, most commonly IOP responses. To date, no review has systematically mapped the field across multiple clinically relevant dimensions, such as incidence, progression, disease severity, and quality of life.

Given the heterogeneity in patient labeling, intervention protocols and outcome definitions, as well as the limited number of randomized trials, a quantitative meta-analysis is currently not appropriate. Instead, a scoping review provides a suitable methodological framework to comprehensively map the existing evidence, identify dominant outcome domains, and highlight underexplored areas relevant to clinical decision-making in this emerging field. The general purpose of this scoping review is to provide the first structured mapping of PA and exercise in POAG.

To achieve this, the scoping review was divided into four research objectives: *prevalence and incidence* in POAG, *disease progression* in POAG, *disease severity* in POAG, and *quality of life* in POAG. This review is conducted in line with the methodological framework for scoping studies (Arksey and O’Malley, 2005).

## Methods

### Protocol and registration

Prior to conducting this review, a protocol was published on Open Science Framework (Schuhmann et al., 2024). The scoping review was conducted according to the Arksey and O’Malley framework (Arksey and O’Malley, 2005; Munn et al., 2018) and reported according to the Preferred Reporting Items for Systematic reviews and Meta-Analyses extension for Scoping Reviews (PRISMA-ScR) (Tricco et al., 2018).

### Eligibility criteria

Studies were included if they investigated patients with primary open-angle glaucoma (POAG), including both normal-tension (NTG) and high-tension glaucoma (HTG). Eligible exposures comprised physical activity and related constructs (e.g., physical activity and physical inactivity, screen time, exercise training, endurance and resistance training, and cardiorespiratory fitness). Comparators were not restricted. Studies were required to report at least one of the following outcomes: (i) ophthalmological markers of disease severity or progression, including functional (e.g., perimetry) and structural parameters derived from imaging such as optical coherence tomography (OCT); (ii) local ocular risk factors (e.g., intraocular pressure, ocular blood flow, ocular perfusion pressure); (iii) systemic risk factors (e.g., inflammation, oxidative stress, vascular structure and function, brain-derived neurotrophic factor (BDNF)); or (iv) vision-related quality of life assessed by patient-reported outcome measures (e.g., NEI VFQ-25, DEQ-5, OSDI, GAL-9). Randomized controlled trials, non-randomized interventional studies, and observational studies were eligible.

For a study to be included, it was sufficient if at least one of the outcomes was reported.

We excluded studies focusing on types of glaucoma other than POAG, animal studies, publications not available in English or German, non-original data (e.g., reviews, editorials), case reports, and qualitative studies. During the iterative screening process, two further exclusion criteria were defined: (i) studies investigating the impact of glaucoma on physical activity (rather than the reverse), and (ii) studies assessing only the acute effects of a single bout of exercise, as our intention was to evaluate longer-term associations or interventions involving physical activity and glaucoma outcomes.

### Information sources

The following databases were searched: *MEDLINE via PubMed, Embase via Elsevier, and SPORTDiscus via EBSCOhost*.

### Search and search management

An information specialist and VS collaboratively developed and refined the search strategy. The search includes text words and database-specific subject headings related to POAG, physical activity, and their variations (last search date: 13/06/2024). The strategy was translated using the Polyglot Search Translator (Clark et al., 2020) and peer-reviewed by a second information specialist. The inception date was defined as the earliest date available in each database and the search was conducted with no restrictions on time or language. The comprehensive electronic search strategies for *MEDLINE (via PubMed*), *EMBASE (via Elsevier)* and *Sportdiscus (via EBSCOhost)* are listed in the **Supplementary Materials**. All search results were imported into Citavi. The Covidence software (https://www.covidence.org/) was used for deduplication, title and abstract screening, full-text screening, and data extraction.

### Selection of sources of evidence

The screening process in Covidence was independently performed by two researchers (VS, JK) and followed a two-stage approach. In the first stage, all articles were assessed based on their title and abstract, and in the second stage, on their full text. Inconsistencies were resolved by consensus between both researchers. In cases of disagreement, a third independent researcher (HH) served as an adjudicator.

### Data charting process

Data extraction was conducted using a data-charting form developed by the lead reviewer (VS) to define the variables of interest. The design of this table was informed by the research aim, ensuring alignment with the objectives of the scoping review. Given the inclusion of both cross-sectional and longitudinal data, the tables were adapted to meet the specific methodological requirements of this approach. Each table (**Tables 1**–**3**) includes information on study name, period, design, and location, sample size, demographics, as well as relevant outcome parameters such as PA/exercise, POAG or disease progression definition, and a summary of the main results.

**Table 1:** Associations between physical activity and exercise in primary open-angle glaucoma: prevalence and incidence.

Author(s)	Study name and timeframe	Study design and aim	Location and ethnicity	Sample size	Population	Physical activity/exercise	Diagnosis of glaucoma	Main findings
Williams (2009)	National Runners’ Health Study (1991-1994)	Prospective, epidemiologic cohort study Assess dose-response relationship of vigorous PA or CRF to the risk for incident glaucoma	USA White (95.1%), Hispanic (2%), Asian (1.2%), Black (0.7%), Native American (0.4%), or mixed ethnicity (0.6%)	N = 29,739	- Adult male runners - Mean age = not reported - Sex male = 100% (95.1% White) - Mean FU = 7.7 years ± 1.8 years - Incidence of glaucoma = 200	PA: running distance per week (self-reported) CRF: 10km performance (self-reported)	Self-reported	- 37% per m/s increment in a 10-km race performance (dose-response relationship) = less glaucoma incidence (RR: 0.633, 95% CI [0.456-0.882]) - 5% per km/d run at baseline (dose-response relationship) = less glaucoma incidence (RR: 0.949, 95% CI [0.902-0.997])
Kawakami et al. (2020)	Niigata wellness study (2001-2002)	Prospective, cohort study Association between MPF and incidence of glaucoma	Japan Asian (100%)	N = 27,051	- Adults ≥ 20–87 years - Median age = 50 years - Sex male = 68% - Median FU = 5.8 years - Glaucoma incidence = 303	MPF: 5 different tests (grip strength, vertical jump, single-leg balance, forward bending, and whole-body reaction time) → MPF index calculation	Self-reported	- MPF index (dose-response relationship) = less glaucoma incidence (HR: 0.64, 95% CI [0.46-0.8])
Meier et al. (2018)	Aerobics Center Longitudinal Study (1987-2005)	Prospective, observational study Association of PA and fitness with incident glaucoma	USA White (68.8%), Black (0.6%), Others (2.1%), Not reported (28.5%)	N = 9,519	- Adults ≥ 40–81 years - Mean age = 50 years - Sex female = 19% - FU = mean 5.7 ± 4.3 years) - Glaucoma development = 128	PA: leisure-time activities (self-reported) CRF: maximal treadmill test	Self-reported	- Active and fit individuals had the lowest risk of incident glaucoma (HR: 0.49, 95% CI [0.31-0.79])
Tseng et al. (2020)	NHANES (2005-2006)	Retrospective, cross-sectional study Association between exercise intensity and glaucoma	USA White (77.4%), Black (9.5%), Mexican American (5.4%), Other (7.6%)	N = 1,387	- Adults ≥ 40 years - Mean age = 55.7 years, - Sex male = 50% - Prevalence of glaucoma = 75 (5.5%)	PA: monitor worn for 7 days (objective) and questionnaire on exercise intensity (subjective)	Glaucoma prevalence: - Rotterdam criteria - Grading of optic disc images by trained ophthalmologists	- Increase in PA intensity = less glaucoma prevalence (OR: 0.97, 95% CI [0.95-0.99] for Rotterdam; OR: 0.90, 95% CI [0.85-0.95] using disc image grading) - Each 10-minute increase in MPA and MVPA per day = less glaucoma prevalence (OR: 0.66, 95% CI [0.50-0.88] for MPA; OR: 0.64, 95% CI [0.49-0.84] for MVPA) - Standing/walking vs. sitting = less glaucoma prevalence (OR: 0.42, 95% CI [0.25-0.70]) - VPA (vs. no VPA) = less glaucoma prevalence (OR: 0.05, 95% CI [0.01-0.56]) - Self-reported moderate daily activity (vs. no activity) = less glaucoma prevalence (OR: 0.05; 95% CI [0.01-0.56]) - No associations between overall self-reported level of activity and glaucoma
De La Cruz et al. (2021)	NHANES (2005-2008)	Cross-sectional study Association of ideal cardiovascular health (Life’s Simple 7) and the prevalence of glaucoma	USA White (76.1%), Black (9.5%), Mexican American (5.9%), Other (8.5%)	N = 6,118	- Adults ≥ 40 years - Mean age = 57 years - Sex male = 47% - Prevalence of glaucoma = 8.1%	PA: number of MVPA performed over the past 30 days (self-reported)	- Self-reported - Retinal imaging	- 1-unit increase in LS7 scores = less glaucoma prevalence (OR: 0.94, 95% CI [0.88-0.99])
Seo and Lee (2022)	KNHANES (2008-2012)	Retrospective, cross-sectional study Association between exercise and glaucoma and association between exercise and IOP levels	Korea Asian (100%)	N = 6,528	- Men ≥ 40 years - Mean age = 54.16 years - Sex male = 100% - Prevalence of glaucoma = 0.9%	Exercise: questionnaire IPAQ-SF (self-reported)	- Health examination survey - Ophthalmic examination based on ISGEO criteria - IOPmax (measured by ophthalmologist)	Exercise and glaucoma prevalence: - MVPA = less glaucoma prevalence (OR: 0.233, 95% CI [0.085-0.637]) - MVPA vs. no exercise = lower risk of glaucoma (OR: 0.338, 95% CI [0.117-0.974]) - Walking vs. no exercise = lower risk of glaucoma (OR: 0.373, 95% CI [0.159-0.877]) - Exercise intensity (dose-response relationship) = less glaucoma prevalence - Highest prevalence of glaucoma in no exercise group (2.0%) - Lowest prevalence of glaucoma in VPA group (0.6%) Exercise and IOP: - IOPmax was higher with increasing exercise intensity - Vigorous intensity exercise group: higher IOPmax
Zhou et al. (2021)	Jiangxi, China (2018)	Cross-sectional questionnaire survey Investigate the prevalence of glaucoma and its related factors	China Asian (100%)	N = 5,385	- Adults ≥ 40 years - Mean age = not reported (highest count in age group 50 years) - Sex male = 41.3% - Prevalence of glaucoma = 78 (1.4%)	PA: standardised questionnaire (self-reported)	Self-reported	- Regular exercise (≥3 d/w vs. <3 d/w) = less glaucoma prevalence (OR: 0.292, 95% CI [0.132-0.642]) - No statistical difference in the prevalence of glaucoma between the sexes (males (1.3%) and females (1.6%))
Fujita et al. (2023)	JMDC Claims Database (2005-2020)	Retrospective, observational cohort study Association between lifestyle habits and development of glaucoma	Japan Asian (100%)	N = 3,110,743	- Adults - Mean age = 44.4 years, - Sex male = 61.7%, - Mean FU = 2058 days - Glaucoma development = 39,975	PA/exercise: health questionnaires (self-reported)	Incidence of glaucoma = having at least one dispensing record of anti-glaucoma eye drops	- Daily walking for 1h = higher glaucoma prevalence (OR: 1.14, 95% CI [1.11-1.16]) - Exercising > 30min twice a week = less glaucoma presence (OR: 0.92, 95% CI [0.90-0.95])
Madjedi et al. (2023)	UK Biobank (2006-2010)	Cross-sectional, observational study Association between PA and glaucoma and related traits (IOP + inner retinal thickness)	England White (91.3%) and non-White (8.7%)	N (self-reported or accelerometer-derived PA and IOP) = 94,206 and 27,777 N (macular inner retinal OCT measurements) = 36,274 and 9,991 N (glaucoma status) = 86,803 and 23,556	- Adults ≥ 37–70 years (subset glaucoma status) - Mean age (baseline) = 56.6 ± 8.1 years (self-reported PA) and 56.5 ± 7.9 years (accelerometer-derived PA) - Sex male = 47.7% - Prevalence of glaucoma = 1.7%	PA: questionnaire – IPAQ (self-reported) and accelerometer (measured for 7 days)	- Diagnosis of glaucoma/history of glaucoma surgery/laser therapy (self-reported) - ICD code for glaucoma (linked hospital records)	- High overall level of self-reported PA = higher IOP (+0.08mmHg, 95% CI [0.02-0.14] – not replicated in the accelerometer data) - Higher overall levels and greater time spent in higher levels (self-reported PA: +0.18µm, 95% CI [0.04-0.31] and accelerometer-derived PA: +0.11µm, 95% CI [0.03-0.18]) = improved OCT measurements of inner retinal thickness (thicker mGCIPL – dose-response relationship) - Higher overall habitual levels of PA and greater duration of time spent in PA = no association with glaucoma status - Higher overall habitual levels of PA and greater duration of time spent in PA = positively associated with thicker mGCIPL and very modestly higher IOP
Lin et al. (2017)	KNHANES (2008-2011)	Population-based, cross-sectional study Association between exercise patterns and glaucoma prevalence	South Korea Asian (100%)	N = 11,246	- Adults ≥ 40 years - Mean age = 58.07 (glaucoma) - Sex female = 44.7% (glaucoma) - Glaucoma development = 336	PA: questionnaire (type/intensity, duration, frequency) (self-reported)	- Defined by ISGEO criteria	- Vigorous exercise 7 d/w compared with 3 d/w = increased glaucoma prevalence (OR: 3.33, 95% CI [1.16-9.54]) - High-intensity exercise compared with moderate-intensity exercise = increased glaucoma prevalence (OR: 1.55, 95% CI [1.03-2.33]) - Other exercise parameters (frequency of moderate exercise, walking, muscle strength and flexibility training, or total minutes of exercise per week) and the prevalence of glaucoma = no association - Sub-analyses (gender): - Vigorous exercise (7 d/w) = increased glaucoma prevalence (men: OR: 6.05, 95% CI [1.67-21.94] but not in women OR: 0.96, 95% CI [0.23-3.97]) (men = ↑; women = no association) - Exercise intensity and glaucoma prevalence in men = U-shaped association - Low intensity vs. moderate intensity: OR: 1.71, 95% CI [1.09-2.69]; high-intensity vs. moderate intensity: OR: 2.19, 95% CI [1.25-3.85]) → Increased glaucoma prevalence in low intensity compared to moderate intensity (subgroup: men) → Increased glaucoma prevalence in high-intensity men compared to moderate intensity (subgroup: men)
Wang et al. (2019)	Beijing Eye Study (2011)	Cross-sectional study Association between the amount of PA and the prevalence of glaucoma	China Asian (100%)	N = 3,031	- Adults ≥ 50–93 years - Mean age = 64.6 ± 9.8 years - Sex male = 43.4% - Prevalence glaucoma = not reported	PA: questionnaire (self-reported)	- Defined by ISGEO criteria	- No association between higher PA score and the prevalence of glaucoma

Overview of the key characteristics of included studies for the association of physical activity/exercise and prevalence/incidence of glaucoma; CI, confidence interval; CRF, cardiorespiratory fitness; d/w, day per week; FU, follow-up; HR, hazard ratio; ICD, International Classification of Diseases; IOP, intraocular pressure; IOPmax, maximal intraocular pressure; IPAQ, International Physical Activity Questionnaire; IPAQ-SF, International Physical Activity Questionnaire Short Form; ISGEO, International Society of Geographical and Epidemiological Ophthalmology; JMDC, Japan Medical Data Centre; KNHANES, Korean National Health and Nutrition Examination Survey; LS7, Life’s Simple 7; mGCIPL, macular ganglion cell-inner plexiform layer; mmHg, millimeters of mercury; MPA, moderate physical activity; MPF, muscular and performance fitness; MVPA, moderate-to-vigorous physical activity; N, sample size; NHANES, National Health and Nutrition Examination Survey; OCT, optical coherence tomography; OR, Odds ratio; PA, physical activity; RR, relative risk; UK, United Kingdom; USA, United States of America; VPA, vigorous physical activity; µm, micrometer; Color coding was used to distinguish the direction of the observed effects (grayscale was used for no/mixed/negative association).

**Table 2:** Associations between physical activity and exercise in primary open-angle glaucoma: disease progression.

Author(s)	Study name and timeframe	Study design and aim	Location and ethnicity	Sample size	Population	Physical activity/exercise	Definition of glaucoma progression	Main findings
Lee et al. (2019)	Johns Hopkins Hospital (2009-2011)	Observational, longitudinal study Association between PA levels and rate of VF loss in glaucoma	USA White (68%) and non-White (32%)	N = 141	- Adults ≥ 60–80 years - Mean age = 64.9 years - Sex male = 20% - Mean baseline eye MD = -6.6 dB (SD, 8.4%)	PA: accelerometer-derived (average steps/day, minutes of non-sedentary activity and minutes of MVPA – worn for 7 days)	Physician-diagnosed glaucoma	- Average rate of VF loss (-0.49 dB/year to -0.04 dB/year) - VF decrease in reference group = -0.33 dB/year (95% CI [-0.38-0.28]) - Each incremental increase of 1000 steps/day = slower VF sensitivity loss over time (-0.007 dB/year) - Each incremental increase of 30-minutes non-sedentary activity/day = slower VF sensitivity loss over time (-0.007 dB/year) - Each 10-minute increase in MVPA = slower annual rate of VF decline (-0.003 dB/year)
Yokota et al. (2016)	University of Fukui Hospital (2014-2015)	Retrospective cohort study Examine the relationship between self-reported habitual exercise and VF defect progression	Japan Asian (100%)	N = 24 (11 exercise group; 13 non-exercise group	- Adults (age range not reported) - Mean age = 69 years - Sex male = 37.5%	PA: questionnaire –further classified into two groups (self-reported)	Physician-diagnosed glaucoma (from clinical records)	- Higher IOP = lower odds for VF defect progression (OR per 1mmHg increase: 0.44, 95% CI [0.11-0.92]) - Habitual exercise = lower odds for VF defect progression (OR: 0.04, 95% CI [0.0003-0.70])
Pan et al. (2020)	Wenzhou Glaucoma Progression Study (2017-2018)	Cross-sectional study Association between exercise habits and VF loss progression in POAG patients	China Asian (100%)	N = 98	- Adults ≥ 18 years, - Mean age = 61.45 years (non-progressive group), 67.22 years (progressive group) - Sex male = 53.52%, 44.44%	PA: accelerometer-derived (calories burned/day, LPA time/day, MPA time/day, VPA time/day, VVPA time/day, MVPA time/day, and step counts – worn for 7 days)	- Defined as open angles on gonioscopy - Glaucomatous optic disc changes - Repeatable VF defects - Above changes in the absence of any other identifiable cause	- 10 mins of MVPA and progressive VF damage (lower OR for VF damage progression: 0.82, 95% CI [0.73-0.92]) - aMAP and progressive VF damage (lower OR for VF damage progression: 0.96, 95% CI [0.94-0.98]) - Male and progressive VF damage (lower OR for VF damage progression: 0.67, 95% CI [0.48-0.96]) - Age and progressive VF damage (increased OR for VF damage progression: 1.06, 95% CI [1.03-1.08]) - SE and progressive VF damage (increased OR for VF damage progression: 1.14, 95% CI [1.07-1.22]) - IOP-lowering medications and progressive VF damage (increased OR for VF damage progression: 1.54, 95% CI [1.16-2.05])

Overview of the key characteristics of included studies for the association of physical activity and exercise and disease progression of glaucoma; aMAP, average mean arterial pressure; CI, confidence interval; dB, decibel; IOP, intraocular pressure; LPA, light physical activity; MD, mean deviation; mmHg, millimeters of mercury; MPA, moderate physical activity; MVPA, moderate to vigorous physical activity; N, sample size; OR, Odds ratio; PA, physical activity; POAG, primary open-angle glaucoma; SD, standard deviation; SE, spherical equivalent; VF, visual field; VPA, vigorous physical activity; VVPA, very vigorous physical activity; Color coding was used to distinguish the direction of the observed effects (grayscale was used for no/mixed/negative association).

**Table 3:** The effects of physical activity and exercise in primary open-angle glaucoma: randomized controlled trials.

Author(s)	Study name and timeframe	Study aim	Location and ethnicity	Sample size	Population	Outcome variables	Diagnosis of glaucoma	Intervention type	Main findings
Hecht et al. (2019)	Department of Ophthalmology, Edith Wolfson Medical Center (2016-2017)	Examine the potential of lifestyle and behavior changes in POAG as add-on treatment and assess short-term cumulative effect on IOP and illness perception	Israel White/top Eastern (100%)	N = 19 (intervention n = 10; control group with continued standard therapy n= 9)	- Adults ≥ 18 years - Mean age: 69 years - Sex male = 37%	Clinical examination: - IOP Questionnaires: - Brief IPQ - Questionnaire about lifestyle and behavioral preferences (baseline and one-month FU) (self-reported) Lifestyle interventions: - Exercise - Diet - Sleep - BMI	- Defined by the combination of a characteristic cupping of the optic disc - Presence of a consistent glaucomatous VF defect - Open anterior chamber angle	Exercise: - 30 min of moderate aerobic PA per day - No strength exercise - No headstands - No swimming googles	Primary outcome (change in IOP after one month): - Treatment group: IOP ↓ by 1.0 mmHg (from 17.5 ± 4.3 – 16.5 ± 4.7 mmHg) - Control group: IOP ↓ by 0.7 mmHg (from 16.8 ± 4.7 – 16.1 ± 6.2 mmHg) - No significant differences in the IOP change between the two groups Secondary outcome (illness perception): - In 3 items (feeling of control, symptoms, and concern about illness) = treatment group significantly improved
Ibrahim and Elbeltagi (2019)	Department of Physical Therapy for Burn and Plastic Surgery (2019)	Evaluate the potential of upper limb resisted exercises as add-on treatment to lower IOP in POAG	Egypt White/top Eastern (100%)	N = 27 (intervention & control group)	- Adults ≥ 40–50 years - Mean age: 47 years - Sex male = 63%	Clinical examination: - IOP Resistance strength testing: - 1RM of biceps brachii	- Physician-diagnosed patients from ophthalmology outpatient clinic	Exercise: - Resistance exercises for the upper limb 3x/week for 4 weeks - Progressive resistance exercise from sets at 40% 1RM to 60% 1RM	Primary outcome (change in IOP): - Treatment group: IOP (mean reduction = 14.97%) ↓ - Control group: IOP (mean reduction = 1.35%) ↓ - Significant difference in pre- and post-treatment values of IOP between treatment and control group
Bhojane et al. (2022)	Department of Ophthalmology Nashik, Maharashtra (2022)	Assess the influence of short- and long-term aerobic exercise on OPP and IOP in POAG	India White/top Eastern (100%)	N = 61 (intervention and control group)	- Adults ≥ 20–70 years - Mean age = 49.2 years - sex male = 51.6%	Clinical examination: - Heart rate - BP - IOP - Adverse outcomes Exercise capacity (watts): - Maximum exercise power (30s) at baseline (on a cycle ergometer)	- Characteristic glaucomatous VF defect - Localised defects of the neuroretinal rim or RNFL - Asymmetry between eyes ≥ 0.2, or cup-to-disc ratio > 0.6 depicting glaucomatous optic neuropathy - Subjects using prostaglandin analogue locally	Short-term effects: - Cycle exercise at moderate and high intensity at 20% and 60% Wmax (10 and 5min) Long-term effects: - Daily jogging for 30min for 3 months at least 20x/month (targeted HR-intensity assessed by sports watch)	Short-term effects: - Lower IOP after 20% Wmax cycling for 10min (mean IOP reduction = 2.88) - Lower IOP after 60% Wmax cycling for 5 min (mean IOP reduction = 5.97) - Reduced IOP related to gender, exercise intensity, and baseline IOP) - Increased OPP after 20% Wmax cycling for 10min (mean OPP improvement = 2.38) - Increased OPP after 60% Wmax cycling for 5min (mean OPP improvement = 5.31) Long-term effects: - Exercise group = IOP ↓ - Control group: no change in IOP Success rate (feasibility): - Short-term: 100% - Long-term: 82% (treatment group)
Ma et al. (2022)	Beijing University of Chinese Medicine (2022)	Investigate change pattern of OPP and IOP after short-term and long-term aerobic exercise	China Asian (100%)	N = 100 (intervention and control group)	- Adults ≥ 22–67 years - Mean age: 50 years - Sex male = 51%	Clinical examination: - IOP - BP - HR - Adverse outcomes Exercise capacity: - Exercise watts	- Defined by the presence of glaucomatous optic neuropathy - Characteristic glaucomatous VF defect	Short-term aerobic exercise: - 10 min cycling exercise at 20% Wmax; 5min of cycling exercise at 60% Wmax Long-term aerobic exercise: - Regular jogging exercise for 30min for 3 months at least 20x/month	Short-term effects: - Lower IOP after 20% Wmax cycling for 10min (mean IOP reduction = 2.6) - Lower IOP after 60% Wmax cycling for 5 min (mean IOP reduction = 5.97) - Higher IOP at baseline = greater IOP reduction after exercise; higher HR during exercise = greater IOP reduction after exercise - Increased OPP after 20% Wmax cycling for 10min (mean OPP improvement = 2.42) - Increased OPP after 60% Wmax cycling for 5min (mean OPP improvement = 5.31) Long-term effects: - Exercise group = IOP ↓ - Control group: no change in IOP Success rate (feasibility): - Short-term: 100% - Long-term: 82% (treatment group)

Overview of the key characteristics of included studies for randomized controlled trials examining the role of physical activity and exercise in glaucoma; BMI, Body Mass Index; BP, blood pressure; FU, follow-up; HR, heart rate; IOP, intraocular pressure; IPAQ, International Physical Activity Questionnaire; IPQ, Illness Perception Questionnaire; mmHg, millimeters of mercury; N, sample size; OPP, ocular perfusion pressure; POAG, primary open-angle glaucoma; 1RM, one repetition maximum; RNFL, retinal nerve fibre layer; VF, visual field; Wmax, maximum watts; Color coding was used to distinguish the direction of the observed effects (grayscale was used for no/mixed/negative association).

Two reviewers (VS, JK) independently charted the data, compared their findings, and iteratively refined the data-charting form. Discrepancies in data extraction were identified and resolved through discussion between the two reviewers, with unresolved cases adjudicated by a third reviewer (HH). While discrepancies were not formally quantified, consensus was reached for all included studies according to the eligibility criteria.

### Synthesis of results

The evidence presented addresses the research aim of this scoping review. As our aim was to identify the currently known body of evidence on the role of PA and exercise in glaucoma patients (POAG), we tabulated the key study characteristics and all necessary metrics in line with the research objectives using numbers and percentages. Afterwards the results were narratively summarized.

**Figure 1:**
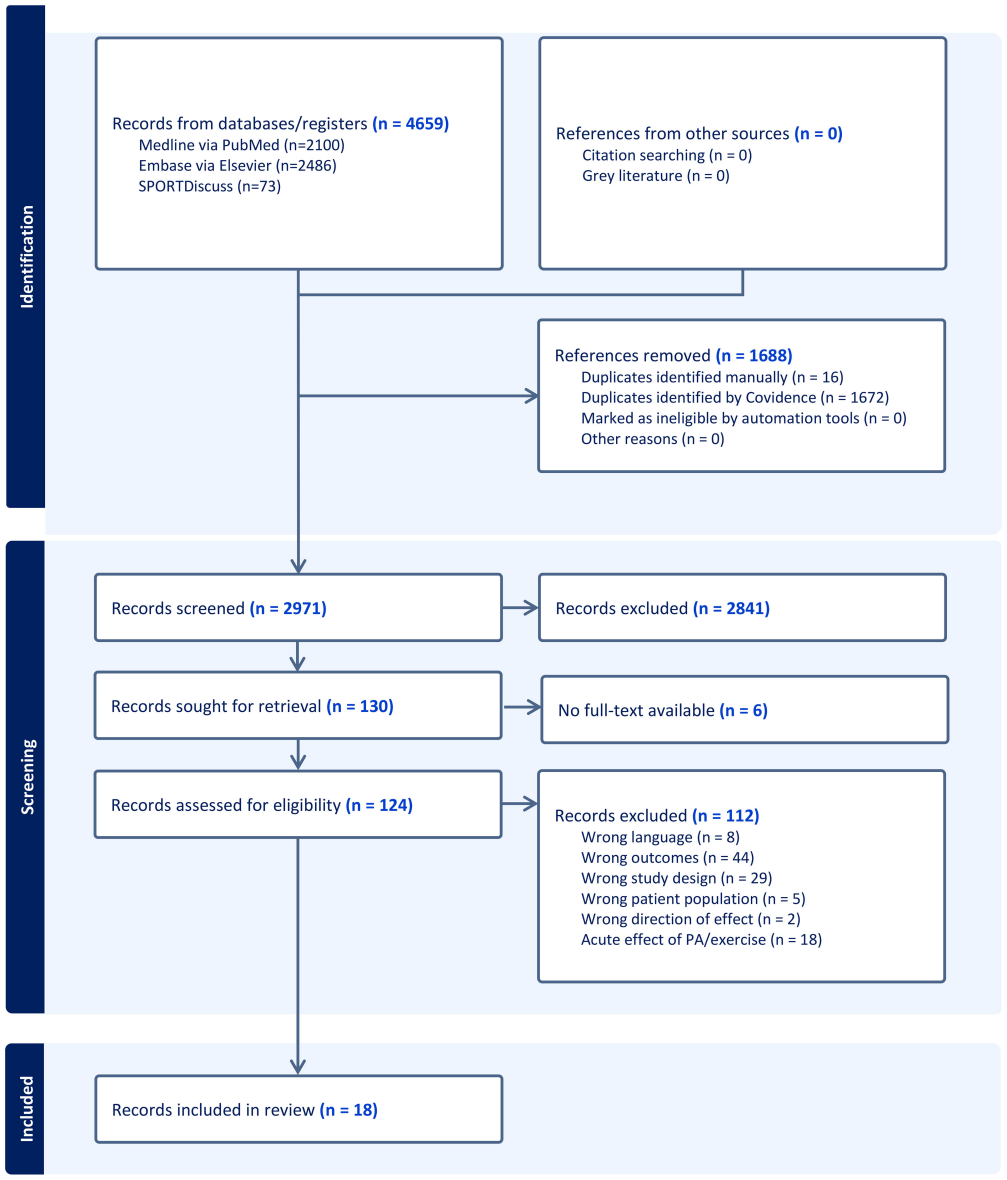
Flow chart of the identification, screening and inclusion process.

The tables are organized according to the specific objectives addressed in this review. The original plan was to create a separate table for each research objective, if data was already available. Additionally, a separate table (**Table 3**) is dedicated exclusively to RCTs. Within each table, studies are arranged chronologically, with the most recent study listed last. Furthermore, studies are categorized based on the direction of the observed effect, distinguishing between those reporting a positive association and those indicating no association, mixed association or a negative association between physical activity/exercise and glaucoma. Color coding was used to categorize the direction of the observed effects (grayscale was used for no/mixed/negative association). A flowchart based on the PRISMA guidelines shows the number of studies at each stage of the screening process (**Figure 1**).

## Results

The literature search yielded 4,659 articles, and after deduplication, 2,971 unique records remained. After title and abstract screening, we obtained 130 studies for full-text screening, of which 18 studies were eligible for data charting. The studies were divided into observational studies (n=14) (Williams, 2009; Yokota et al., 2016; Lin et al., 2017; Meier et al., 2018; Lee et al., 2019; Wang et al., 2019; Kawakami et al., 2020; Pan et al., 2020; Tseng et al., 2020; De La Cruz et al., 2021; Zhou et al., 2021; Seo and Lee, 2022; Fujita et al., 2023; Madjedi et al., 2023) and RCTs (n=4) (Hecht et al., 2019; Ibrahim and Elbeltagi, 2019; Bhojane et al., 2022; Ma et al., 2022). The results are discussed below according to the respective research objectives. No studies were found on the effect of PA and exercise on POAG severity (objective 3) or quality of life in POAG patients (objective 4).

A visual representation of the identification, selection, and evaluation of relevant studies is provided in the flow chart below (**Figure 1**).

### Prevalence and incidence

The evidence on the association between PA and POAG prevalence and incidence (**Table 1**) is mixed, reflecting both protective and inconsistent findings across observational studies. The majority of studies reported a consistent favorable association, with vigorous PA, higher cardiorespiratory fitness (CRF), greater muscular performance fitness, and active lifestyle behaviors linked to reduced glaucoma incidence (Williams, 2009; Meier et al., 2018; Kawakami et al., 2020). Moderate-to-vigorous PA (MVPA), frequent weekly exercise, and better cardiovascular health scores (e.g., Life’s Simple 7) were also associated with lower glaucoma prevalence (Tseng et al., 2020; De La Cruz et al., 2021; Zhou et al., 2021), even in a dose-dependent pattern (Seo and Lee, 2022).

Conversely, other studies revealed heterogeneous, null, or even adverse associations. An increased glaucoma prevalence associated with daily walking (1 hour), alongside a protective effect of structured exercise (>30 minutes), was reported in one study, suggesting that exercise type and intensity may differentially affect POAG prevalence while (Fujita et al., 2023). A UK Biobank study reported no significant link between PA and glaucoma prevalence but observed positive associations between PA and retinal structure, implying potential early neuroprotective effects (Madjedi et al., 2023). Additionally, one study reported higher glaucoma prevalence in men engaging in both low and highly vigorous daily PA, suggesting a possible U-shaped relationship. The according odds ratios were widely ranged (OR: 6.05, 95% CI [1.67-21.94]) and only significant in men. Several other parameters, including walking, strength training, and total PA duration, showed no associations in this study (Lin et al., 2017). Lastly, one study found no significant relationship between PA scores and glaucoma prevalence (Wang et al., 2019).

### Disease progression

Studies on the relation between PA and POAG are depicted in **Table 2**. Three studies consistently showed that higher PA levels are associated with slower visual field (VF) deterioration (Yokota et al., 2016; Lee et al., 2019; Pan et al., 2020).

One study reported a dose-response relationship, with greater steps per day and time spent in MVPA were linked to slower VF sensitivity loss (Lee et al., 2019). This finding was backed-up by another publication, showing each 10-minute increase in MVPA reduced the odds of VF progression. The study also identified male sex and higher mean arterial pressure to be protective, whereas older age, myopia, and IOP-lowering medication use increased the risk of progression (Pan et al., 2020). Finally, similar results were reported for lower odds and progression of VF with regular exercise, although an unexpected inverse relationship was found between IOP and VF deterioration, highlighting the complex association between PA, IOP, and disease progression (Yokota et al., 2016).

### Randomized controlled trials

**Table 3** shows included studies with RCT-design. Among the four included RCTs, three demonstrated positive effects of PA in POAG, while one reported a mixed result.

Three RCTs reported significant physiological benefits of PA in POAG patients, particularly on IOP and ocular perfusion pressure (OPP). One study showed significant IOP reduction with resistance training (Ibrahim and Elbeltagi, 2019), and another study demonstrated that both short- and long-term aerobic exercise reduced IOP and increased OPP, with greater effects at higher intensities and sustained benefits over time. Interestingly, the feasibility of structured exercise interventions in POAG patients is proven by high adherence rates (Bhojane et al., 2022). These findings were further confirmed, supporting PA as a feasible and effective adjunct in glaucoma management (Ma et al., 2022).

No significant IOP reduction after a one-month lifestyle intervention was found in the fourth RCT, although participants did report improved illness perception (Hecht et al., 2019).

### Disease severity and quality of life

POAG is a chronic, progressive disease with considerable psychological strain on patients, proven by the lower scores on vision-related quality of life questionnaires (Huang et al., 2020). Notably, our results highlight a significant gap in research exploring the relationship between PA/exercise and both disease severity and quality of life. No studies addressed disease severity or quality of life. Evidence is concentrated on IOP and short-term outcomes, while clinically relevant domains such as disease severity and patient-reported outcome measures remain largely unexplored.

## Discussion

Unlike previous reviews that primarily focused on IOP responses or specific exercise interventions, this scoping review systematically mapped evidence across multiple clinically relevant domains in POAG. While IOP changes are well documented, several vascular, neuroprotective pathways, and systemic mechanisms with the potential to counteract the incidence, progression, and severity of glaucoma are also described (Wisløff et al., 2007; Conte et al., 2014; Ramos et al., 2015; Zhu et al., 2018; Chu-Tan et al., 2022; van Eijgen et al., 2024). This review suggests that PA and exercise might have the potential to reduce both the incidence and progression in POAG patients beyond reducing IOP and improving ocular perfusion pressure (OPP).

### Potential mechanisms linking physical activity and glaucoma

Proposed mechanisms for local retinal and known systemic effects of PA and exercise beyond IOP changes primarily fall within the following major groups: microvascular endothelial dysfunction; ocular perfusion pressure; mitochondrial health and oxidative stress; chronic inflammation and neuronal degeneration (Zhu et al., 2018; Gracitelli et al., 2019; Chu-Tan et al., 2022).

First, exercise is known to improve vascular and endothelial function primarily through enhanced nitric oxide bioavailability and reduced arterial stiffness (Ashor et al., 2014; Green and Smith, 2018; Ferreira et al., 2024). These adaptations may improve ocular blood flow and its autoregulation, which are critical given that impaired microcirculation of the optic nerve head has been implicated in glaucoma pathophysiology. Improved vascular function may therefore mitigate ischemic stress and increase the resilience of retinal ganglion cells.

Second, physical activity influences OPP, a key determinant of optic nerve head perfusion defined by the interaction between systemic blood pressure and intraocular pressure (Schmidl et al., 2011; Costa et al., 2014; Gracitelli et al., 2019). During exercise, OPP typically increases due to sympathetic-mediated elevations in systemic blood pressure alongside transient reductions in intraocular pressure, likely driven by decreased episcleral venous pressure and fluid dynamic shifts (Gracitelli et al., 2019). Acute exercise has consistently been shown to transiently reduce intraocular pressure (Gildea et al., 2024), thereby further contributing to this increase in OPP (Ma et al., 2022). The effect of increased OPP on ocular circulation is complex, while choroidal blood flow is relatively pressure-passive and may increase with higher perfusion pressures, retinal circulation is tightly autoregulated in healthy individuals. In glaucoma, however, impaired vascular autoregulation may alter this response, potentially contributing to instability in ocular blood flow. Chronic exercise may further improve vascular regulation and systemic blood pressure control, which could enhance OPP stability over time. These effects may be particularly relevant in normal-tension glaucoma, where vascular dysregulation is considered a central mechanism.

Third, exercise promotes mitochondrial function and reduces oxidative stress through increased biogenesis and improved metabolic efficiency, while reducing the accumulation of reactive oxygen species (Kawamura and Muraoka, 2018; Chu-Tan et al., 2022). Given the high metabolic demand of retinal ganglion cells and the central role of mitochondrial dysfunction in glaucoma, these adaptations may contribute to enhanced cellular resilience and reduced neurodegeneration. From a mechanistic perspective, excessive exercise could theoretically increase oxidative stress or provoke hemodynamic fluctuations, potentially affecting vulnerable retinal tissues and ultimately contributing to the development of glaucoma (Izzotti et al., 2006; Kruk et al., 2015). However, extensive evidence from cardiovascular and neurological research indicates that higher exercise intensities are often associated with greater systemic benefits, including anti-inflammatory, antioxidant, and vascular adaptations (Swain and Franklin, 2006; Wisløff et al., 2007; Ramos et al., 2015; Hanssen et al., 2017; K B S et al., 2024).

Fourth, exercise exerts systemic anti-inflammatory and neuroprotective effects. Chronic physical activity is associated with reduced levels of pro-inflammatory markers and increased expression of neurotrophic factors such as brain-derived neurotrophic factor, which may support the survival and function of retinal ganglion cells (Chrysostomou et al., 2016; Chu-Tan et al., 2022).

Overall, the IOP-independent vascular, metabolic and neuroprotective mechanisms are likely interdependent and may help slow glaucomatous damage. However, the current evidence remains largely indirect, and further mechanistic and interventional studies are needed to confirm disease-modifying effects and to clarify the causal pathways linking physical activity, exercise and glaucoma.

### Prevalence and incidence

For the dimension of prevalence and incidence, most studies reported beneficial associations between specific characteristics of PA and glaucoma-related outcomes, whereas others showed mixed, null, or even unfavorable associations. Overall, patterns related to PA intensity, duration, and frequency appeared to influence glaucoma risk, with sustained and vigorous exercise behaviors, as well as higher cardiorespiratory fitness (CRF), being associated with lower glaucoma prevalence (Meier et al., 2018; Tseng et al., 2020; Zhou et al., 2021). Some studies demonstrated a dose–response relationship, indicating progressively lower glaucoma prevalence with increasing PA or CRF levels, highlighting CRF as a potentially important modifier of ocular health (Williams, 2009).

Beyond aerobic capacity, muscular strength and overall physical performance were also linked to reduced glaucoma risk (Kawakami et al., 2020), suggesting that general physical fitness may exert broader protective effects. Synergistic associations were observed in individuals who were both physically active and physically fit, who exhibited the lowest glaucoma risk (Meier et al., 2018). Interestingly, while vigorous PA was sometimes associated with higher IOP, it was also linked to lower glaucoma prevalence, implying that potential benefits may occur through IOP-independent mechanisms (Seo and Lee, 2022). Proposed pathways include systemic anti-inflammatory effects, improved vascular function, and reduced neuroinflammation (Tribble et al., 2021; Chu-Tan et al., 2022). In line with this, better overall cardiovascular health, as reflected by higher Life’s Simple 7 scores (De La Cruz et al., 2021), was associated with lower glaucoma likelihood, reinforcing the concept of systemic-ocular interactions (Flammer et al., 2013; Dascalu et al., 2021; Choi et al., 2023).

However, several studies reported conflicting findings. Some large population-based investigations found no association between PA and glaucoma prevalence (Wang et al., 2019), or only structural retinal benefits without clear reductions in IOP or glaucoma risk. These findings suggest that the short-term IOP-lowering effects of exercise may not necessarily translate into long-term reductions in glaucoma risk (Madjedi et al., 2023). Nonetheless, associations between higher PA and thicker macular ganglion cell–inner plexiform layer thickness support the hypothesis of neuroprotective effects independent of IOP (Chrysostomou et al., 2016; Chieffi et al., 2017). Experimental evidence suggests that PA may enhance retinal perfusion (Alnawaiseh et al., 2017), suppress inflammatory pathways (Kuehn et al., 2006), and increase neurotrophic factors such as brain-derived neurotrophic factor (BDNF) (Chrysostomou et al., 2016).

In contrast, some observational data indicated heterogeneous or even adverse associations. Inconsistent findings were found for structured PA (>30 min, 2x/week) and daily walking for one hour, where the first was linked to reduced glaucoma risk and the second to increased risk. This may either suggest that PA type, frequency, and intensity might have differential effects, or might point to low external validity of the results, given the likely bias of the glaucoma label used in this study (Fujita et al., 2023).

One study suggested a higher glaucoma prevalence among individuals, particularly men, engaging in high-intensity PA compared with those performing moderate-intensity activity, leading to the hypothesis of a U-shaped relationship between exercise intensity and glaucoma risk (Lin et al., 2017). It is noteworthy that the reported odds ratios are unusually high (OR: 6.05, 95% CI [1.67–21.94]). Furthermore, the presumed U-shaped relationship is based on a single observational study with self-reported PA measurements, inaccurate intensity classifications, and potentially biased definitions of glaucoma, which should therefore be interpreted with caution.

### Disease progression

For disease progression, regular exercise has been consistently associated with reduced odds of VF loss in glaucoma. Yokota et al. reported a strong association between regular PA and reduced VF defect progression. Interestingly, higher IOP was paradoxically linked to a lower risk of VF defect progression. One possible explanation for this may be clinical decision making or the result of treatment itself. Patients with higher IOP levels might receive more aggressive IOP-lowering therapy or severe (thus progressive) glaucoma patients might be more aggressively treated and therefore end up with lower IOPs (Yokota et al., 2016). Additionally, greater engagement in MVPA was associated with reduced odds of progressive VF damage, with each 10-minute increase in MVPA conferring additional benefits in individuals with POAG (Pan et al., 2020). Moreover, glaucoma progression is commonly attributed to elevated IOP, vascular dysregulation, and impaired ocular perfusion (Heijl et al., 2002; Liu et al., 2016). Exercise not only reduces IOP but also enhances ocular perfusion, improving choroidal blood flow, ocular perfusion pressure, and ocular blood pressure, which may help explain the association between higher PA and a reduced risk of progressive glaucomatous VF damage. Even a clear dose-response relationship was observed, where incremental increases in daily steps and non-sedentary time were associated with slower VF sensitivity decline (Lee et al., 2019). However, the optimal type and measure of PA most strongly linked to ocular health remain unclear, though evidence suggests that even light activity may be beneficial. Yet, the observed effects on VF loss appear modest. Importantly, data from models evaluating VFs prior to activity assessment suggest that progressive VF damage may lead to reduced PA, indicating potential bidirectionality in this relationship. The association between faster VF decline and lower activity levels, even in models limited to the 5 years preceding activity assessment, further supports the possibility that VF deterioration may contribute to PA restriction (Lee et al., 2019). Greater levels of PA have been associated with statistically significant, albeit very modest, reductions in the rate of VF loss in POAG, with observed effects ranging from –0.007 to –0.003 dB/year (Lee et al., 2019). While the magnitude of this effect is small, the potential neuroprotective properties of PA and exercise may positively influence the underlying pathophysiological processes affecting the optic nerve. These findings highlight PA as a modifiable behavioral factor in POAG management and support the need for further longitudinal and mechanistic research to better understand its therapeutic potential and the concrete magnitude of this effect.

### Randomized controlled trials

For RCTs, most articles found beneficial effects of exercise interventions on glaucoma and related traits. A RCT by Bhojane and colleagues demonstrated both short- and long-term benefits of aerobic cycling (Bhojane et al., 2022). In the short term, both low-intensity (20% Wmax) and moderate-intensity (60% Wmax) cycling reduced IOP, with more pronounced effects observed at higher intensity. Additionally, OPP increased post-exercise, particularly following moderate-intensity sessions. In the long term, participants who maintained regular exercise exhibited sustained IOP reductions, whereas no significant change was seen in the control group. High adherence rates (100% in the short term and 82% in the long term) further underscore the feasibility of implementing such interventions in clinical practice. A study by Ma et al. confirmed the beneficial effects of similar exercise interventions on both IOP and OPP, thereby strengthening the existing evidence base (Ma et al., 2022). The results of the two studies are consistent with the widely established fact that dynamic exercise can lower IOP and improve OPP (Price et al., 2003; Natsis et al., 2009; Risner et al., 2009; Zhu et al., 2018; van Eijgen et al., 2024; Zhang et al., 2024). Ibrahim and Elbeltagi extended the findings on the effects of aerobic exercise on IOP. The authors found a significant improvement in IOP in the intervention group, which underwent four weeks of resisted exercises, compared to no changes in the control group. Possible mechanisms for these findings could be increased blood lactate and osmolarity, where the rise in blood osmolarity causes water outflux from the eye, contributing to IOP reduction (Buckingham and Young, 1986; Price et al., 2003). Additionally, exercise increases adrenergic hormone release due to increased stimulation of the release of epinephrine and norepinephrine from the adrenal medulla. These hormones increase cyclic adenosine monophosphate levels, which reduce aqueous humor production, thereby lowering IOP (Qureshi et al., 1997; Martin et al., 1999). Enhanced trabecular outflow via adrenaline may be another mechanism, where elevated adrenaline may improve trabecular meshwork outflow and reduce aqueous humor formation, contributing to IOP decline (Wang et al., 2002). Lastly, exercise may improve pulsatile ocular blood flow and thereby decreasing IOP levels, potentially protecting against glaucoma-related damage (Price et al., 2003).

Furthermore, the interplay between PA and exercise, blood pressure (BP) regulation (including autoregulation), and OPP may substantially influence the prevalence and progression of POAG. Evidence suggests a bimodal, U-shaped relationship between BP and disease progression, with both hypotension and hypertension being associated with adverse outcomes such as reduced retinal nerve fiber layer and ganglion cell thickness, particularly in the absence of adequate autoregulation (Van Eijgen et al., 2024). PA lowers BP through multiple physiological mechanisms, including enhanced vascular function, reduced arterial stiffness, and improved weight regulation (Farpour-Lambert et al., 2009; Börjesson et al., 2016; Kobayashi, 2025). Moreover, PA may normalize and reduce BP variability, thereby offering additional protective effects (Hanssen et al., 2022; Esmailiyan et al., 2023). The combination of increased nitric oxide bioavailability, reduced oxidative stress and improved arterial compliance, may enhance the autoregulatory capacity and thereby ameliorate the eye’s ability to buffer BP fluctuations, ultimately slowing disease progression (Van Eijgen et al., 2024; Kobayashi, 2025). However, research in this area remains limited, and the precise mechanisms underlying the complex relationship between POAG and BP are not yet fully understood.

While the majority of studies included in this review support a beneficial effect of PA on IOP, findings from one RCT introduce a nuanced perspective (Hecht et al., 2019). In their study, a one-month exercise intervention did not lead to a significant reduction in IOP. However, the intervention group demonstrated improved illness perception, suggesting psychological benefits independent of measurable changes in ocular physiology even in such a short-term intervention. Likely, the intervention was too short to induce long-lasting effects on IOP. These findings underscore the multifaceted benefits of PA, extending beyond IOP or disease modulation. Improvements in illness perception may foster greater engagement in self-management behaviors, improve treatment adherence, and reduce the psychological burden of chronic conditions such as glaucoma (Prince et al., 2007). This aligns with broader literature on the mental health benefits of exercise, emphasizing the role of PA in holistic glaucoma care (Deslandes et al., 2009; Smith and Merwin, 2021; Eather et al., 2023). Together, these three RCTs provide consistent and converging evidence that both resistance and aerobic exercise may serve as a valuable adjunct to conventional glaucoma treatment for lowering IOP and improving OPP, with strong feasibility for clinical integration, given the high adherence rates observed. Furthermore, higher intensity appears to confer greater benefits, although all exercise types may be effective.

### Influence of type of exercise on glaucoma-related outcomes

Interpreting the current findings in this field of research is complicated by the considerable variation that exists across studies in terms of how physical activity is defined and measured. To facilitate comparison, physical activity domains can be broadly categorized as *aerobic exercise* (e.g., running, cycling, swimming), *strengthening or resistance exercise* (e.g., weightlifting, resistance band training) (Garcia-Hermoso et al., 2023), *vigorous activity* (typically ≥6 metabolic equivalents, such as high-intensity interval training or fast running) (Lopez et al., 2020), and *sustained or habitual activity* (regularly accumulated physical activity over time, such as daily walking or recreational sports participation) (Chastin et al., 2019). However, these classifications were not consistently applied across studies, and exercise intensity was frequently assessed using heterogeneous self-reported or device-based methods (Warner et al., 2022; Gildea et al., 2024). This variability likely contributes to inconsistencies in reported glaucoma-related outcomes and limits comparability across studies.

When stratified by type of exercise, more nuanced modes of exercise emerge. *Aerobic and mild-to-moderate intensity activity* were most consistently associated with favorable outcomes, including lower glaucoma prevalence and slower VF progression (Williams, 2009; Meier et al., 2018; Tseng et al., 2020; Zhou et al., 2021), potentially reflecting beneficial vascular and metabolic adaptations. In contrast, *vigorous or high-intensity exercise* showed more heterogeneous associations (Lin et al., 2017; Fujita et al., 2023), which may relate to transient fluctuations in intraocular pressure and ocular perfusion dynamics. Evidence on *resistance training* remains limited and inconclusive, with some studies suggesting short-term physiological changes without clear long-term impact on glaucoma outcomes.

In addition, it is important to distinguish between acute physiological responses to a single exercise bout and long-term training adaptations (Rivera-Brown and Frontera, 2012). Acute exercise induces transient changes, including reductions in intraocular pressure and increases in ocular perfusion pressure, which may influence short-term ocular hemodynamics but do not necessarily translate into sustained effects (Risner et al., 2009; Gildea et al., 2024). In contrast, habitual physical activity promotes longer-term adaptations, such as improved endothelial function, vascular autoregulation, and metabolic efficiency, that are more likely to confer neuroprotective benefits (Chu-Tan et al., 2022; Gildea et al., 2024). Age may further modify these relationships, given age-related differences in vascular function and glaucoma susceptibility (Boutouyrie et al., 2021; Zhang et al., 2021). This distinction is critical when interpreting heterogeneous findings across studies and their relevance for glaucoma progression.

Current evidence does not allow firm conclusions regarding optimal physical activity and exercise. Achieving beneficial glaucoma-related outcomes likely depends on prescription of exercise type, intensity, and duration, underscoring the need for more standardized future research in the field.

### Limitations and future research

A limitation of this work is its hybrid format between a scoping and a systematic review. The current evidence base in this field is insufficient to allow for a thorough risk of bias assessment or meta-analysis. Nonetheless, this review synthesizes the available literature to outline the potential impact of PA and exercise in POAG and identifies key methodological gaps that should guide future research.

Across all studies investigating the relationship between PA and POAG, whether addressing prevalence, incidence, progression, or conducted as RCTs, two major limitations consistently compromise the quality and interpretability of the evidence. First, glaucoma status was often based on self-reported diagnoses without clinical verification, introducing substantial risk of misclassification bias. Second, there was a lack of detailed clinical data, with key ocular and systemic parameters (e.g., visual field indices, OCT metrics, central corneal thickness, diurnal intraocular pressure variation, and cardiorespiratory fitness) frequently unmeasured or unaccounted for, limiting both mechanistic understanding and comparability across studies.

Several recurring methodological limitations further weaken the strength, interpretability, and generalizability of the existing evidence. Most prevalence and incidence studies rely on cross-sectional designs and self-reported PA, with inconsistent definitions and assessments limited to single time points, factors that introduce bias and preclude causal inference.

Progression studies are affected by selection bias (e.g., exclusion of advanced cases), small sample sizes, and insufficient detail on PA type, intensity, and context. The use of retrospective or cross-sectional designs limits insights into temporal relationships.

RCTs are constrained by short intervention durations, small sample sizes, and incomplete outcome reporting, often focusing solely on short-term IOP changes while neglecting long-term effects or functional outcomes.

To clarify the therapeutic potential of PA in glaucoma care, future studies should employ longitudinal, adequately powered designs with clinically verified glaucoma diagnoses and standardized, objective PA assessments across diverse populations.

## Conclusion

This scoping review highlights a growing but methodologically heterogeneous body of literature examining the relationship between PA, exercise, and POAG. While several studies report favorable associations between higher PA levels and lower POAG prevalence, incidence, and progression, findings, particularly for prevalence and incidence, remain partly inconsistent. Randomized controlled trials are scarce and frequently limited by small sample sizes, short follow-up periods, and heterogeneous outcome measures.

Importantly, no original study has comprehensively evaluated the impact of PA on disease severity or vision-related quality of life using validated instruments, underscoring substantial evidence gaps. Furthermore, the effects of exercise on retinal microvascular function, ocular blood flow regulation, and systemic cardiovascular contributors such as blood pressure variability and vascular dysregulation, mechanisms especially relevant in NTG, remain insufficiently investigated.

Given the multifactorial and vascular components of glaucoma pathophysiology, focusing solely on IOP as a target outcome appears insufficient to fully capture disease dynamics. PA and exercise may influence IOP-independent mechanisms; however, current evidence does not yet allow definitive conclusions regarding causality, optimal exercise dosing, or clinical applicability.

Overall, current evidence suggests a potential role of PA and exercise in POAG, although, the evidence base remains limited, particularly regarding disease severity and patient-reported outcomes. Well-designed, adequately powered, and longer-term randomized trials incorporating comprehensive ophthalmological, vascular, and quality-of-life endpoints are needed before specific clinical recommendations can be made.

## Data Availability

The original contributions presented in the study are included in the article/**Supplementary Material**. Further inquiries can be directed to the corresponding author.
