# Supplementary material for: Physical activity and exercise in primary open-angle glaucoma: a scoping review
Source: Front Physiol. 2026 Jul 15;17:1834649. doi: 10.3389/fphys.2026.1834649 (PMC13414203; doi:10.3389/fphys.2026.1834649)
Supplement: Supplementary file 1 [file SupplementaryFile1.docx]

# Supplementary material

### Supplement 1: Search strategy

The comprehensive electronic search strategy for all databases *MEDLINE database via PubMed*, *Embase via Elsevier,* and *SPORTDiscus via EBSCOhost* are described below. The search string includes text words and database-specific subject headings related to POAG, physical activity, and their variations. The searches were conducted as outlined in the section “Information sources”, with no restrictions on time or language. To ensure relevance, all animal studies were excluded. For clarity and ease of interpretation, search terms have been color-coded (i.e. highlighting MeSH terms); this has no other meaning.

#### Medline via PubMed search string:

Number of records retrieved by the search: 2100

Search conducted on: 13/06/2024

("Glaucoma"[Mesh:NoExp] OR "Glaucoma, Open-Angle"[Mesh:NoExp] OR "Low Tension Glaucoma"[Mesh] OR glaucoma*[Tiab] OR POAG[tiab])

AND ("Recreation"[Mesh:NoExp] OR "Hunting"[Mesh] OR "Sports"[Mesh] OR "Exercise"[Mesh:NoExp] OR "Cool-Down Exercise"[Mesh] OR "Exergaming"[Mesh] OR "Gymnastics"[Mesh] OR "Muscle Stretching Exercises"[Mesh] OR "Physical Conditioning, Human"[Mesh] OR "Post-Exercise Recovery"[Mesh] OR "Preoperative Exercise"[Mesh] OR "Warm-Up Exercise"[Mesh] OR "Rehabilitation"[Mesh:NoExp] OR "Cardiac Rehabilitation"[Mesh] OR "Telerehabilitation"[Mesh] OR "Dance Therapy"[Mesh] OR "Recreation Therapy"[Mesh] OR "exercise therapy"[MeSH Terms] OR "Aquatic Therapy"[Mesh] OR "Sedentary Behavior"[Mesh] OR "Life Style"[Mesh:NoExp] OR "Screen Time"[Mesh] OR exercis*[tiab] OR "physical activ*"[tiab] OR "physically activ*"[tiab] OR "physical inactiv*"[tiab] OR "physically inactiv*"[tiab] OR sedentary[tiab] OR "Screen Time*"[tiab] OR rehabilitation[tiab] OR telerehabilitation[tiab] OR endurance[tiab] OR Stamina[tiab] OR Fitness[tiab] OR Aerobic[tiab] OR Sport*[tiab] OR "Aerobics"[tiab] OR "AcroYoga"[tiab] OR "aquatic"[tiab] OR "Archery"[tiab] OR "Badminton"[tiab] OR "Ball*"[tiab] OR baseball[tiab] OR basketball[tiab] OR "Biking"[tiab] OR Bicycling[tiab] OR "Bobsledding"[tiab] OR "Bodybuilding"[tiab] OR "Bowling"[tiab] OR "Boxing"[tiab] OR "Breakdancing"[tiab] OR "Bouldering"[tiab] OR "Bowling"[tiab] OR "Calisthenics"[tiab] OR "Canoeing"[tiab] OR "Capoeira"[tiab] OR "Caving"[tiab] OR "Cricket"[tiab] OR "CrossFit"[tiab] OR "Curling"[tiab] OR "Cycling"[tiab] OR "Danc*"[tiab] OR "exergaming"[tiab] OR "Fencing"[tiab] OR "Fishing"[tiab] OR football[tiab] OR "Freerunning"[tiab] OR "Geocaching"[tiab] OR "Golf"[tiab] OR "Gymnastics"[tiab] OR HIIT[tiab] OR "Hike*"[tiab] OR "Hockey"[tiab] OR "Hunting"[tiab] OR "Jogg*"[tiab] OR "Jumping"[tiab] OR "Karate"[tiab] OR "Kayaking"[tiab] OR "Kickboxing"[tiab] OR "Kiteboarding"[tiab] OR "Lacrosse"[tiab] OR "Martial arts"[tiab] OR mountaineering[tiab] OR "Obstacle course racing"[tiab] OR "Orienteering"[tiab] OR "Parkour"[tiab] OR "Pilates"[tiab] OR "Powerlifting"[tiab] OR "Rafting"[tiab] OR "Riding"[tiab] OR "Rock climbing"[tiab] OR "Rollerblading"[tiab] OR "Rowing"[tiab] OR "Rugby"[tiab] OR "Runn*"[tiab] OR "Run"[tiab] OR "Sailing"[tiab] OR "Shooting"[tiab] OR "Skateboarding"[tiab] OR "Skating"[tiab] OR "Skiing"[tiab] OR "Slacklining"[tiab] OR "Snorkeling"[tiab] OR "Snowboard*"[tiab] OR "Soccer"[tiab] OR "Squash"[tiab] OR "Surfing"[tiab] OR "Swimm*"[tiab] OR "Tai Chi"[tiab] OR "Tennis"[tiab] OR "Tobogganing"[tiab] OR "Training" [tiab] OR "Trapeze"[tiab] OR "Trekking"[tiab] OR "Triathl*"[tiab] OR "Ultimate Frisbee"[tiab] OR volleyball[tiab] OR "Wakeboarding"[tiab] OR "Walking"[tiab] OR "Water skiing"[tiab] OR "Weightlifting"[tiab] OR "Weight lifting"[tiab] OR "Windsurfing"[tiab] OR "Wrestling"[tiab] OR "Yoga"[tiab] OR "Zumba"[tiab])

NOT ("animals"[MeSH Terms] NOT "humans"[MeSH Terms])

#### EMBASE via Elsevier search string:

Number of records retrieved by the search: 2486

Search conducted on: 13/06/2024

('glaucoma'/de OR 'glaucomatous optic neuropathy'/de OR 'intraocular hypertension'/de OR 'low tension glaucoma'/de OR 'open angle glaucoma'/de OR 'primary glaucoma'/de OR glaucoma*:ti,ab OR POAG:ti,ab)

AND ('recreation'/de OR 'dancing'/de OR 'sport'/exp OR 'human activities'/de OR 'exercise'/exp OR 'exercise recovery'/de OR 'rehabilitation'/de OR 'athletic rehabilitation'/de OR 'functional assessment'/de OR 'functional training'/de OR 'heart rehabilitation'/de OR 'muscle training'/de OR 'telerehabilitation'/de OR 'dance therapy'/de OR 'recreational therapy'/de OR 'aquatic therapy'/de OR 'physical activity'/exp OR fitness/de OR 'lifestyle'/de OR 'sedentary lifestyle'/de OR Physical inactivity/de OR 'screen time'/de OR (exercis* OR 'physical activ*' OR 'physically activ*' OR 'physical inactiv*' OR 'physically inactiv*' OR sedentary OR 'Screen Time*' OR rehabilitation OR telerehabilitation OR endurance OR Stamina OR Fitness OR Aerobic OR Sport* OR Aerobics OR AcroYoga OR aquatic OR Archery OR Badminton OR Ball* OR baseball OR basketball OR Biking OR Bicycling OR Bobsledding OR Bodybuilding OR Bowling OR Boxing OR Breakdancing OR Bouldering OR Bowling OR Calisthenics OR Canoeing OR Capoeira OR Caving OR Cricket OR CrossFit OR Curling OR Cycling OR Danc* OR exergaming OR Fencing OR Fishing OR football OR Freerunning OR Geocaching OR Golf OR Gymnastics OR HIIT OR Hike* OR Hockey OR Hunting OR Jogg* OR Jumping OR Karate OR Kayaking OR Kickboxing OR Kiteboarding OR Lacrosse OR 'Martial arts' OR mountaineering OR 'Obstacle course racing' OR Orienteering OR Parkour OR Pilates OR Powerlifting OR Rafting OR Riding OR 'Rock climbing' OR Rollerblading OR Rowing OR Rugby OR Runn* OR Run OR Sailing OR Shooting OR Skateboarding OR Skating OR Skiing OR Slacklining OR Snorkeling OR Snowboard* OR Soccer OR Squash OR Surfing OR Swimm* OR 'Tai Chi' OR Tennis OR Tobogganing OR Training OR Trapeze OR Trekking OR Triathl* OR 'Ultimate Frisbee' OR volleyball OR Wakeboarding OR Walking OR 'Water skiing' OR Weightlifting OR 'Weight lifting' OR Windsurfing OR Wrestling OR Yoga OR Zumba):ti,ab)

NOT ((animal/de OR animal experiment/exp OR nonhuman/de) NOT (human/exp OR human experiment/de))

NOT ([conference abstract]/lim OR [letter]/lim OR [editorial]/lim OR [preprint]/lim)

#### Sportdiscus via EBSCOhost search string:

Number of records retrieved by the search: 73

Search conducted on: 13/06/2024

(DE "Glaucoma" OR (TI "glaucoma*" OR AB "glaucoma*") OR (TI "POAG" OR AB "POAG"))

AND (DE "SPORTS" OR DE "AGE & sports" OR DE "AMATEUR sports" OR DE "AQUATIC sports" OR DE "BALL games" OR DE "BASEBALL" OR DE "COMBAT sports" OR DE "CONTACT sports" OR DE "CROSS-training (Sports)" OR DE "DISC golf" OR DE "ENDURANCE sports" OR DE "EXTREME sports" OR DE "GAELIC games" OR DE "HOCKEY" OR DE "INDIVIDUAL sports" OR DE "RECREATIONAL sports" OR DE "SPORT for all" OR DE "SPORTS for older people" OR DE "TEAM sports" OR DE "WINTER sports" OR DE "RECREATION" OR DE "OUTDOOR recreation" OR DE "RECREATION for older people" OR DE "RECREATIONAL sports" OR DE "LEISURE" OR DE "HUNTING" OR DE "EXERCISE" OR DE "ABDOMINAL exercises" OR DE "AEROBIC exercises" OR DE "ANAEROBIC exercises" OR DE "AQUATIC exercises" OR DE "ARM exercises" OR DE "BACK exercises" OR DE "BREATHING exercises" OR DE "BUTTOCKS exercises" OR DE "CALISTHENICS" OR DE "CHAIR exercises" OR DE "CHEST exercises" OR DE "CIRCUIT training" OR DE "COMPOUND exercises" OR DE "COOLDOWN" OR DE "EXERCISE for middle-aged persons" OR DE "EXERCISE for older people" OR DE "EXERCISE video games" OR DE "FOOT exercises" OR DE "GYMNASTICS" OR DE "HAND exercises" OR DE "HIP exercises" OR DE "ISOKINETIC exercise" OR DE "ISOLATION exercises" OR DE "ISOMETRIC exercise" OR DE "ISOTONIC exercise" OR DE "KNEE exercises" OR DE "LEG exercises" OR DE "MUSCLE strength" OR DE "PILATES method" OR DE "QI gong" OR DE "RUNNING" OR DE "SHOULDER exercises" OR DE "STRENGTH training" OR DE "TAI chi" OR DE "TREADMILL exercise" OR DE "WHEELCHAIR workouts" OR DE "YOGA" OR DE "PHYSICAL training & conditioning" OR DE "ACTIVE recovery" OR DE "ALTITUDE training" OR DE "ANAEROBIC training" OR DE "ARCHERY training" OR DE "BADMINTON training" OR DE "BASE training (Exercise)" OR DE "BASEBALL training" OR DE "BASKETBALL training" OR DE "BICYCLE racing training" OR DE "BODYBUILDING" OR DE "BOWLING training" OR DE "CONTINUOUS training (Exercise)" OR DE "CONTRAST training (Physical training & conditioning)" OR DE "CROSS-training (Sports)" OR DE "CYCLING training" OR DE "DANCE training & conditioning" OR DE "ENDURANCE sports training" OR DE "FENCING training" OR "FOOTBALL training" OR DE "FUNCTIONAL training" OR DE "GOLF training" OR DE "GYMNASTICS training" OR DE "HANDBALL training & conditioning" OR DE "HIKING training & conditioning" OR DE "HOCKEY training" OR DE "INTERVAL training" OR DE "ISOLATION exercises" OR DE "LACROSSE training & conditioning" OR DE "LONG slow distance training" OR DE "MARTIAL arts training" OR DE "MOUNTAINEERING training" OR DE "PACE training" OR DE "PERIODIZATION training" OR DE "PERSONAL training" OR DE "PRACTICE (Sports)" OR DE "REPETITION training" OR DE "RESISTANCE training" OR DE "ROWING training" OR DE "RUNNING training" OR DE "SKI training" OR DE "SOCCER training" OR DE "SPEED endurance training" OR DE "SQUASH training" OR DE "STRENGTH training" OR DE "SURFING training" OR DE "SWIMMING training" OR DE "TABLE tennis training & conditioning" OR DE "TENNIS training" OR DE "TRIATHLON training" OR DE "VOLLEYBALL training" OR DE "WEIGHT training" OR DE "WHEELCHAIR sports training" OR DE "WINTER sports training & conditioning" OR DE "YOGA training & conditioning" OR DE "WARMUP" OR DE "WALKING" OR DE "REHABILITATION" OR DE "MEDICAL rehabilitation" OR (DE "DANCE") OR (DE "RECREATIONAL therapy") OR DE "SEDENTARY lifestyles" OR DE "PHYSICAL fitness" OR DE "SEDENTARY behavior" OR (TI "exercis*" OR AB "exercis*") OR (TI "physical activ*" OR AB "physical activ*") OR (TI "physically activ*" OR AB "physically activ*") OR (TI "physical inactiv*" OR AB "physical inactiv*") OR (TI "physically inactiv*" OR AB "physically inactiv*") OR (TI "sedentary" OR AB "sedentary") OR (TI "Screen Time*" OR AB "Screen Time*") OR (TI "rehabilitation" OR AB "rehabilitation") OR (TI "telerehabilitation" OR AB "telerehabilitation") OR (TI "endurance" OR AB "endurance") OR (TI "Stamina" OR AB "Stamina") OR (TI "Fitness" OR AB "Fitness") OR (TI "Aerobic" OR AB "Aerobic") OR (TI "Sport*" OR AB "Sport*") OR (TI "Aerobics" OR AB "Aerobics") OR (TI "AcroYoga" OR AB "AcroYoga") OR (TI "aquatic" OR AB "aquatic") OR (TI "Archery" OR AB "Archery") OR (TI "Badminton" OR AB "Badminton") OR (TI "Ball*" OR AB "Ball*") OR (TI "baseball" OR AB "baseball") OR (TI "basketball" OR AB "basketball") OR (TI "Biking" OR AB "Biking") OR (TI "Bicycling" OR AB "Bicycling") OR (TI "Bobsledding" OR AB "Bobsledding") OR (TI "Bodybuilding" OR AB "Bodybuilding") OR (TI "Bowling" OR AB "Bowling") OR (TI "Boxing" OR AB "Boxing") OR (TI "Breakdancing" OR AB "Breakdancing") OR (TI "Bouldering" OR AB "Bouldering") OR (TI "Bowling" OR AB "Bowling") OR (TI "Calisthenics" OR AB "Calisthenics") OR (TI "Canoeing" OR AB "Canoeing") OR (TI "Capoeira" OR AB "Capoeira") OR (TI "Caving" OR AB "Caving") OR (TI "Cricket" OR AB "Cricket") OR (TI "CrossFit" OR AB "CrossFit") OR (TI "Curling" OR AB "Curling") OR (TI "Cycling" OR AB "Cycling") OR (TI "Danc*" OR AB "Danc*") OR (TI "exergaming" OR AB "exergaming") OR (TI "Fencing" OR AB "Fencing") OR (TI "Fishing" OR AB "Fishing") OR (TI "football" OR AB "football") OR (TI "Freerunning" OR AB "Freerunning") OR (TI "Geocaching" OR AB "Geocaching") OR (TI "Golf" OR AB "Golf") OR (TI "Gymnastics" OR AB "Gymnastics") OR (TI "HIIT" OR AB "HIIT") OR (TI "Hike*" OR AB "Hike*") OR (TI "Hockey" OR AB "Hockey") OR (TI "Hunting" OR AB "Hunting") OR (TI "Jogg*" OR AB "Jogg*") OR (TI "Jumping" OR AB "Jumping") OR (TI "Karate" OR AB "Karate") OR (TI "Kayaking" OR AB "Kayaking") OR (TI "Kickboxing" OR AB "Kickboxing") OR (TI "Kiteboarding" OR AB "Kiteboarding") OR (TI "Lacrosse" OR AB "Lacrosse") OR (TI "Martial arts" OR AB "Martial arts") OR (TI "mountaineering" OR AB "mountaineering") OR (TI "Obstacle course racing" OR AB "Obstacle course racing") OR (TI "Orienteering" OR AB "Orienteering") OR (TI "Parkour" OR AB "Parkour") OR (TI "Pilates" OR AB "Pilates") OR (TI "Powerlifting" OR AB "Powerlifting") OR (TI "Rafting" OR AB "Rafting") OR (TI "Riding" OR AB "Riding") OR (TI "Rock climbing" OR AB "Rock climbing") OR (TI "Rollerblading" OR AB "Rollerblading") OR (TI "Rowing" OR AB "Rowing") OR (TI "Rugby" OR AB "Rugby") OR (TI "Runn*" OR AB "Runn*") OR (TI "Run" OR AB "Run") OR (TI "Sailing" OR AB "Sailing") OR (TI "Shooting" OR AB "Shooting") OR (TI "Skateboarding" OR AB "Skateboarding") OR (TI "Skating" OR AB "Skating") OR (TI "Skiing" OR AB "Skiing") OR (TI "Slacklining" OR AB "Slacklining") OR (TI "Snorkeling" OR AB "Snorkeling") OR (TI "Snowboard*" OR AB "Snowboard*") OR (TI "Soccer" OR AB "Soccer") OR (TI "Squash" OR AB "Squash") OR (TI "Surfing" OR AB "Surfing") OR (TI "Swimm*" OR AB "Swimm*") OR (TI "Tai Chi" OR AB "Tai Chi") OR (TI "Tennis" OR AB "Tennis") OR (TI "Tobogganing" OR AB "Tobogganing") OR (TI "Training" OR AB "Training") OR (TI "Trapeze" OR AB "Trapeze") OR (TI "Trekking" OR AB "Trekking") OR (TI "Triathl*" OR AB "Triathl*") OR (TI "Ultimate Frisbee" OR AB "Ultimate Frisbee") OR (TI "volleyball" OR AB "volleyball") OR (TI "Wakeboarding" OR AB "Wakeboarding") OR (TI "Walking" OR AB "Walking") OR (TI "Water skiing" OR AB "Water skiing") OR (TI "Weightlifting" OR AB "Weightlifting") OR (TI "Weight lifting" OR AB "Weight lifting") OR (TI "Windsurfing" OR AB "Windsurfing") OR (TI "Wrestling" OR AB "Wrestling") OR (TI "Yoga" OR AB "Yoga") OR (TI "Zumba" OR AB "Zumba"))
